# The Vulvar Immunohistochemical Panel (VIP) Project: Molecular Profiles of Vulvar Squamous Cell Carcinoma

**DOI:** 10.3390/cancers13246373

**Published:** 2021-12-19

**Authors:** Giorgia Garganese, Frediano Inzani, Simona Maria Fragomeni, Giulia Mantovani, Luigi Della Corte, Alessia Piermattei, Angela Santoro, Giuseppe Angelico, Luciano Giacò, Giacomo Corrado, Anna Fagotti, Gian Franco Zannoni, Giovanni Scambia

**Affiliations:** 1 Dipartimento Scienze della Vita e Sanità Pubblica, Sezione Ginecologia e Ostetricia, Università Cattolica del Sacro Cuore, 00168 Rome, Italy; giorgia.garganese@materolbia.com (G.G.); anna.fagotti@policlinicogemelli.it (A.F.); giovanni.scambia@policlinicogemelli.it (G.S.); 2 Gynecology and Breast Care Center, Mater Olbia Hospital, 07026 Olbia, Italy; 3 Unità di Gineco-Patologia e Patologia Mammaria, Fondazione Policlinico Universitario A. Gemelli IRCCS, 00168 Rome, Italy; frediano.inzani@policlinicogemelli.it (F.I.); alessia.piermattei@policlinicogemelli.it (A.P.); angela.santoro@policlinicogemelli.it (A.S.); giuseppe.angelico@policlinicogemelli.it (G.A.); gianfranco.zannoni@policlinicogemelli.it (G.F.Z.); 4 Unità di Ginecologia Oncologica, Fondazione Policlinico Universitario A. Gemelli IRCCS, 00168 Rome, Italy; simona.fragomeni@policlinicogemelli.it (S.M.F.); luigi.dellacorte2@studenti.unina.it (L.D.C.); giacomo.corrado@policlinicogemelli.it (G.C.); 5 Department of Obstetrics and Gynaecology, Gynaecologic Oncology and Minimally-Invasive Pelvic Surgery, International School of Surgical Anatomy, IRCCS Sacro Cuore-Don Calabria Hospital, 37024 Negrar di Valpolicella, Italy; 6 Department of Neuroscience, Reproductive Sciences and Dentistry, School of Medicine, University of Naples Federico II, 80131 Naples, Italy; 7 Bioinformatics Facility Core Research, Gemelli Science and Technology Park, Fondazione Policlinico Universitario A. Gemelli IRCCS, 00168 Rome, Italy; luciano.giaco@policlinicogemelli.it; 8 Dipartimento Scienze della Vita e Sanità Pubblica, Sezione Anatomia Patologica, Università Cattolica del Sacro Cuore, 00168 Rome, Italy

**Keywords:** biostatistics, gynecological cancers, immunohistochemistry, molecular targets, vulvar cancer, squamous cell carcinoma

## Abstract

**Simple Summary:**

This study investigated the immunohistochemical expression of 14 biological markers as potential prognostic/therapeutic factors in vulvar squamous cell carcinoma, comparing 53 node-negative (Group A) and 48 node-positive (Group B) patients. Our results show a significantly higher p16 expression (surrogate of HPV-related tumors) in the vulvar samples of non-metastatic patients. In Group B, PD-L1 positivity and high EGFR expression were found in the vast majority of vulvar and/or nodal specimens. VEGF showed strong/moderate-diffuse expression in almost 14% of all vulvar samples. A mutated p53 and over-expressed PD-L1 showed a significant association with nodal metastasis. Our results support a potential role of immune checkpoint inhibitors and anti-VEGF and anti-EGFR drugs, especially in patients with worse prognosis (metastatic, HPV-independent). A panel including EGFR, VEGF, PDL1, p16, and p53 might be performed routinely in primary tumor and repeated in case of lymph node metastases to identify changes in marker expression.

**Abstract:**

Introduction: The study’s aim was to investigate the immunohistochemical (IHC) expression of biological markers as potential prognostic/therapeutic factors in vulvar squamous cell carcinoma (VSCC). Methodology: A series of 101 patients surgically treated at our center from 2016 to 2020 were retrospectively enrolled: 53 node-negative (Group A) and 48 node-positive (Group B). A total of 146 samples, 101 from primary tumor (T) and 45 from nodal metastases (N), were investigated. The IHC panel included: p16, p53, MLH1, MSH2, MSH6, PMS2, PD-L1, CD3, HER2/neu, ER, PR, EGFR, VEGF, and CD31. The reactions were evaluated on qualitative and semi-quantitative scales. Generalized Linear Model (GLM) and cluster analysis were performed in R statistical environment. A distance plot compared the IHC panel of T with the correspondent N. Results: In Group A: p16-positive expression (surrogate of HPV-dependent pathway) was significantly higher (20.8% vs. 6.2%, *p* = 0.04). In Group B: PD-L1 positivity and high EGFR expression were found, respectively, in 77.1% and 97.9% patients (T and/or N). Overall, p16-negative tumors showed a higher PD-L1 expression (60.9% vs. 50.0%). In both groups: tumoral immune infiltration (CD3 expression) was mainly moderate/intense (80% vs. 95%); VEGF showed strong/moderate-diffuse expression in 13.9% of T samples; CD31, related to tumoral microvessel density (MVD), showed no difference between groups; a mutated p53 and over-expressed PD-L1 showed significant association with nodal metastasis, with Odds Ratios (OR) of 4.26 (CI 95% = 1.14–15.87, *p* = 0.03) and 2.68 (CI 95% = 1.0–7.19, *p* < 0.05), respectively; since all mismatch repair proteins (MMR) showed a retained expression and ER, PR, and HER2/neu were negative, they were excluded from further analysis. The cluster analysis identified three and four sub-groups of molecular profiles, respectively, in Group A and B, with no difference in prognosis. The molecular signature of each N and corresponding T diverged significantly in 18/41 (43.9%) cases. Conclusions: Our results support a potential role of immune checkpoint inhibitors and anti-VEGF and anti-EGFR drugs especially in patients with worse prognosis (metastatic, HPV-independent). A panel including EGFR, VEGF, PDL1, p16, and p53 might be performed routinely in primary tumor and repeated in case of lymph node metastases to identify changes in marker expression.

## 1. Introduction

Although vulvar squamous cell cancer (VSCC) is a rare disease [1,2], in the last decade an increasing number of studies have been focused on widening the knowledge of pathogenetic mechanisms and overcoming the limits of diagnostic and therapeutic options currently available, including imaging work-ups, conservative and reconstructive surgery, and interventional treatments [3].

In particular, the greatest advances have been achieved in the early stages, in terms of higher diagnostic precision [4,5,6,7,8,9,10,11] and extended indications for conservative treatment [12,13].

As regards the management of locally advanced stages, surgical treatment still remains the pivotal option [14], despite often requiring large tissue resections, plastic reconstruction, and careful management of severe morbidity [15,16,17]. However, some new developments have been introduced as effective possible alternative strategies currently available, including radiation or chemo-radiation, but these suffer from the main limitation of non-repeatability over time, in case of recurrence [18,19,20,21].

No significant improvement has been realized in cases of poor response, progression, or distant metastasis. In these cases, few additional options are suitable, including local interventional treatments, systemic chemotherapy, or palliative care, and these unfortunately often show limited results [19,22,23,24,25].

In particular, it is widely documented that in this type of tumor, the response to commonly used chemotherapy agents is suboptimal, showing very weak and provisional results [24,25].

Within this framework, developing the ability to identify specific biomarkers, predicting VSCC behavior and tailoring target-directed therapeutic agents, should be a priority to improve more efficient treatment strategies, especially for advanced stages with poor prognosis [26].

Some progress has already been made in understanding the mechanisms of carcinogenesis, with evidence that VSCC can originate from precursor lesions, according to two different pathogenetic pathways: (a) from the progression of high-grade squamous intraepithelial lesions (HSIL), in a human papilloma virus (HPV)-related pathway; and (b) from the progression of a vulvar intraepithelial neoplasia of differentiated type (dVIN) or, as suggested by some authors, a lichen sclerosus (LS), in a non-HPV-related pathway [3,27,28,29].

Unfortunately, vulvar cancer still remains unaccounted for in terms of a complete genetic and molecular characterization [30], and no relevant marker has yet been clearly identified among the sparse and low-quality data available for prognostic and therapeutic purposes. Therefore, there is an urgent need to define the molecular expression of VSCC in order to identify specific targets for treatments.

After a systematic review of the literature and in the light of the experience of our previous study on the molecular characterization of vulvar Paget’s disease [31,32], we focused our research on four main pathways, considering their influence on carcinogenesis: (1) tumor immune microenvironment, (2) activation of oncogenic growth factor receptors, (3) hormonal environment, and (4) neo-angiogenesis. From each of these areas, we selected the main biological markers, chosen on the basis of their relevance as potential prognostic/therapeutic factors, as already known from the study of squamous tumors in other anatomical regions.

The aim of this study was to investigate the immunohistochemical (IHC) expression of a panel of 14 selected biological markers in squamous cell carcinoma of the vulva.

## 2. Methods

### 2.1. Patients and Study Design

This is a single-institution retrospective study, approved by the Institutional Ethics Committee (Prot. 6996/18. ID: 1932). All consecutive patients with histologically proven invasive squamous cell carcinoma of the vulva referred to our institution from March 2016 to March 2020 were considered eligible. Their surgical and clinical management had been decided on the basis of specific clinical, radiological, and histopathological features, according to current guidelines and after discussion by a dedicated tumor board, supported by the institutional Vulvar Cancer Multi-Disciplinary Team (Vul.Can MDT). Enrolled patients signed a written informed consent. Their clinical data were retrieved from patients’ medical records, collected using Research Electronic Data Capture (REDCap) tool, and managed according to privacy regulations.

Patients were excluded in cases of: recurrent VSCC; non-standard or incomplete surgery on the primary tumor site and inguino-femoral lymph nodes (LNs); paraffin-embedded material that was insufficient for or at risk of being completely consumed by the analyses required by the study; and incomplete clinical data, not allowing assessment of oncologic outcome.

IHC expression of a panel of 14 biological markers was tested on tissue samples from each enrolled patient’s primary tumor site; in cases of metastatic LNs, the IHC panel was additionally tested on the metastatic site.

The IHC panel included: p16, p53, programmed death-ligand 1 (PD-L1), cluster of differentiation 3 (CD3), human epidermal growth factor receptor 2 (HER2/neu), epidermal growth factor receptor (EGFR), estrogen receptor (ER), progesterone receptor (PR), vascular endothelial growth factor (VEGF), CD31, and the four mismatch repair proteins (MMR) known as MSH2, MSH6, MLH1, and PMS2.

Based on the IHC results, patients were subsequently clustered according to their molecular profiles, in order to identify possible subgroups of prognostic/therapeutic relevance.

### 2.2. Sample Processing

An IHC panel was performed on one representative primary tumor block from each vulvar site and repeated on one representative metastatic lymph node site in metastatic cases. Hematoxylin and eosin (HE) stain sections from formalin-fixed paraffin-embedded (FFPE) blocks were reviewed by pathologists in a blinded manner, and the diagnoses were compared. IHC staining of 4-µm sections of FFPE tissue was performed with appropriate primary rabbit anti-human monoclonal antibodies (mAbs). Additional information about the antibodies used in immunohistochemistry are reported in the Supplementary Section (Table S1).

Negative and positive control staining versus reactivity with the mAbs were performed in each series.

### 2.3. Immunohistochemical Interpretation

IHC staining of all slides was scored by two observers. Slides were scored negative if positive internal controls were well-stained.

p16 marker was scored on a semi-quantitative scale and then dichotomized as elsewhere reported [33]. The semi-quantitative scale comprised three categories: negative (score 0) when diffusely weak, “patchy” when nuclear and cytoplasmic staining was detected in less than 50% of tumor cells (score 1), and overexpressed (score 2) when diffuse nuclear positivity of tumor cells, with at least moderate intensity, was present. Tumors reporting score 0 and score 1 were defined as HPV-independent (p16-negative), while tumors reporting score 2 were defined as HPV-associated (p16-positive).

p53 was classified into normal pattern (wild type: score 1) and mutated patterns (null type: score 0 and overexpression: score 2). A wild type pattern was recognized by focal nuclear staining of variable intensity in up to 50% of tumor cells. This pattern should also be observed in the internal controls (stromal cells, lymphocytes, or non-neoplastic epithelium). The overexpression of p53 was evaluated according to the percentage of positive cell nuclei in respect to the negative (wild type) and was considered positive with moderate or strong staining intensity in more than 50% of tumor cells (typically more than 80% of the nuclei in tumor cells were stained). The null pattern refers to the complete absence of staining in tumor cell nuclei, in the presence of wild type staining in the internal control. For statistical purposes, the results of p53 expression were dichotomized into two types: p53-wild type (score 1) and p53-mutated (score 0 and 2).

PD-L1 evaluation was performed for tumor cells but not for tumor-infiltrating immune cells. Tumor cells with circumferential or partial membranous staining were considered positive for PD-L1. Cytoplasmic staining in the absence of membrane signals was recorded as negative. The expression of PD-L1 was scored as a percentage of tumor cells with positive PD-L1 membranous staining of any intensity, and it was considered positive for analysis if greater than or equal to 5% of tumor cells were showing membranous immunoexpression.

The expression of CD3 in T cells was evaluated to determine the presence of tumor-infiltrating lymphocytes (TILs) in the vulvar site. Positive signals were detected using the recommendations provided by the International TILs Working Group 2014 for breast cancer [34]. The protein expression of MMR was considered positive when positive nuclei with mild to strong intensities were counted; it was negative if internal controls (stromal cells and lymphocytic infiltrates) were positive and tumor cells were completely negative. Tumors were classified as MMR-proficient (pMMR) if informative and positive for all four MMR proteins and as MMR-deficient (dMMR) in the absence of expression of at least one of the four proteins.

The EGFR mutation-specific staining was scored based on membrane staining intensity compared to the surrounding non-neoplastic epithelium: 0 = no staining, 1 = faint staining in <10% of tumor cells, 2 = moderate membranous staining; 3 = strong membranous staining. The results with intensity of 0 and 1 were considered as EGFR low (L), and the results with intensity 2 and 3 were defined as EGFR high (H) expression.

HER2/neu staining intensity was evaluated using the ASCO/CAP breast scoring criteria [35].

Tumors with positive ER or PR nuclear staining in a fraction of neoplastic cells ≥1% were defined as ER- or PR-positive, respectively.

The VEGF cytoplasmic expression was evaluated on the basis of the intensity signal (absent: 0; weak: 1; moderate: 2; strong: 3) and the percentage of positive tumor cells. The composite score was calculated considering both the parameters and was classified as negative if the signal was absent, weak, or moderate with intensity <10% of tumor cells and positive if the signal was moderate in ≥10% of tumor cells or strong.

Microvessel density (MVD) was only studied in the primary tumor site with CD31. MVD “hotspots” were counted in three 20× high-power fields within the malignant tumor and in one non-neoplastic section (CTRL). Results were reported as the highest vessel density (MaxMVD) and the mean vessel density (MeanMVD) for each case. The ratio of MeanMVD-to-CTRL for each patient (MVDratio) was calculated.

### 2.4. Statistical Analysis

The Fisher test was used to evaluate the difference between positive and negative lymph node groups of patients with regard to IHC markers expression and patients’ characteristics. Age and infiltration characteristics were evaluated with the Wilcoxon test. For IHC markers, a Generalized Linear Model (GLM) was built in order to evaluate the relationship of the antibody markers to the lymph node positivity outcome. Cluster analysis was performed using the hclust algorithm with a CluMix package version 2.3.1 [36]. The correlation analysis and plot were carried out using corrplot package [37]. All analyses were performed in R statistical environment. Survival analysis and Cox Proportional Hazard model were used to assess the hazard for lymph node status, biomarkers and clusters. Overall survival (OS) was defined as the time from surgical treatment to death from any cause. Disease-free survival (DFS) was defined as the time from surgical treatment to disease recurrence or death from any cause. Statistical significance was considered achieved if the *p*-Value obtained was lower than 0.05.

## 3. Results

### 3.1. Clinico-Pathological Findings

A total of 107 patients affected by a primary invasive vulvar carcinoma were enrolled in the study. After IHC analysis, six patients were excluded because, for technical reasons, additional sections were needed to complete the study, which would have exhausted the paraffin sample; a total of 101 patients were included in the final analysis. Clinical, surgical, and histopathologic features of the two groups are reported in Table 1. The median age at diagnosis was 78 (48–96) years, similar in the two groups. Surgery on the primary tumor site was performed as follows: 30 (29.7%) partial vulvectomies, 49 (48.75%) radical vulvectomies, and 22 (21.8%) ultra-radical vulvectomies (including extra-vulvar neighboring tissues). Inguinal lymph node surgical staging was performed bilaterally in 90.1% and monolaterally 5.9% of cases, as appropriate, for a total of 188 groins surgically treated according to the following procedures: sentinel lymph node (SNB) biopsy in 56 (29.7%) groins; SNB followed by inguinofemoral lymphadenectomy in 79 (42.1%) groins; and radical lymphadenectomy in 53 (28.2%) groins. Lymph node surgical staging was omitted in 4 (4.0%) very elderly (>90 years) patients, being frail, with severe comorbidities, and all having been assessed as clinically node-negative at a careful preoperative workup (ultrasound and PET/CT scan) (4, 8).

At final histology, 53 patients (Group A = 52.5%) had negative lymph nodes and 48 (Group B = 47.5%) were positive. No statistically significant difference was noted between the two groups regarding the site (anterior, central, posterior, and lateral), diameter (median diam. 35 mm, range 4–105), and focality of the primary vulvar lesion. Instead, tumor grade (G) stratification was significantly different in the two groups, G3 being more represented in Group B (14.6% vs. 3.8%; *p* = 0.001), as well as lymphovascular invasion (62.5% vs. 3.8%; *p* < 0.0001). Depth of invasion was also higher in Group B (median = 7 mm vs. 6 mm; *p* = 0.001). Overall, FIGO stage was stratified into 49 (48.5%) Stage I, 4 (4%) Stage II, 43 (42.6%) Stage III, and 5 (4.9%) Stage IV patients.

**Figure 1:**
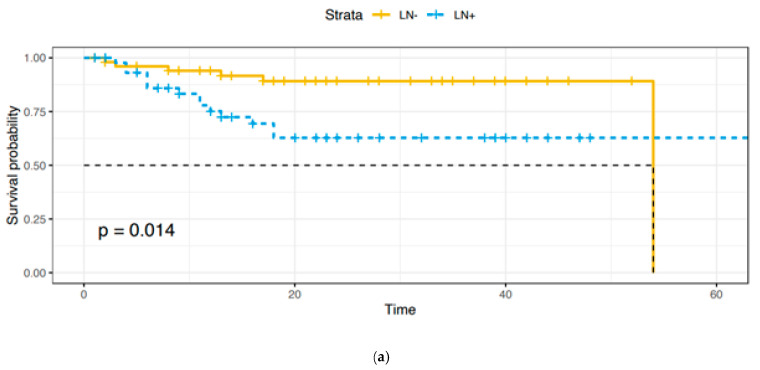

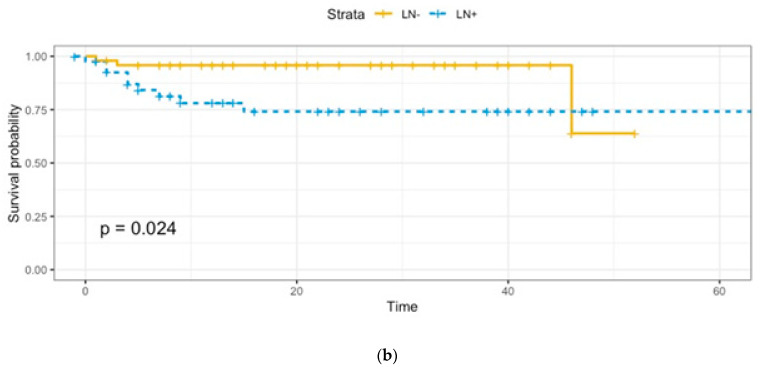
(**a**). Overall survival: Group A vs. Group B. (**b**). Disease-free survival: Group A vs. Group B.

Adjuvant treatments were provided as follows: 53 (91.4%) patients received radiotherapy, 3 (5.2%) received concomitant radio-chemotherapy, and 2 (3.4%) received chemotherapy. A total of 12 patients died of disease, 2 in Group A and 10 in Group B, with a statistically significant difference (*p* = 0.006). As expected, overall survival (OS) and disease-free survival (DFS) were lower in Group B (Figure 1a,b).

### 3.2. Immunohistochemical Results

The results of IHC staining and SISH performed on primary vulvar site (Group A and Group B) and on metastatic LNs (Group B) are summarized in Table 2 and Supplementary Figures S1 and S2.

### 3.3. Oncogenic Growth Factor-Receptors

#### 3.3.1. p16 and p53

Starting from the main biomarkers related to the pathogenesis of vulvar cancer, a prevalence of p16-negative and/or p53-mutated HPV-independent tumors was observed. In details: 87/101 (86.1%) tumors were p16-negative, and 14/101 (13.9%) were p16-positive (HPV-associated); 26/101 (25.7%) tumors were p53-wild type, and 75/101 (74.3%) were p53-mutated. The percentage of p16-positive cancers was lower in the metastatic group (Group B = 6.2%) compared to the non-metastatic group (Group A = 20.8%) (*p* = 0.04). On the other hand, p53-mutated cancers were more frequently observed in the metastatic group (Group B = 83.3%) compared to the non-metastatic group (Group A = 66%) (*p* = 0.06). Among p16-positive tumors (*n* = 14), only three (21.4%) were p53-mutated (double positivity). When p16 was negative (*n* = 87), only 15 primary tumors (17.2%) were p53-wild type (double negativity). Therefore, the vast majority of vulvar neoplasms (83/101 = 82.2%) were p16-positive or p53-mutated in a mutually exclusive way.

#### 3.3.2. EGFR

EGFR-H was found in 42/53 (79.2%) primary tumors from Group A and in 43/48 (89.6%) Group B, with no statistically significant difference. More specifically in group B, a total number of 47/48 (97.9%) patients were EGFR-H in at least one of the two sites of disease (primary tumor and/or metastatic lymph node). A similar distribution of EGFR immunostaining was observed between HPV and non-HPV-related cancers. In detail, EGFR-H tumors were observed in 12/14 (85.7%) HPV-related cancers and 73/87 (83.9%) non-HPV-related cancers.

#### 3.3.3. HER2

In our analysis, an HER2/neu amplification was found in only 2/101 vulvar sites and 3/45 nodal sites (missing data in three cases); therefore, this marker was excluded from all subsequent correlation analyses.

### 3.4. Tumoral Immune Microenvironment

Positive PD-L1 immunostaining was detected in 28/53 (52.8%) primary vulvar cancers from Group A and 32/48 (66.7%) from Group B, with no statistically significant differences. Moreover, 27/45 (60%) metastatic LNs were PD-L1-positive (missing data in three cases). In five metastatic cancers, PD-L1 staining was negative in the vulvar site and positive in the lymph nodes. Overall, 37/48 (77.1%) of patients affected by metastatic disease had a PD-L1-positive tumor in the primitive and/or metastatic site. By contrast, PD-L1 expression was lost in eight metastatic cancers in the nodal site. Among HPV-related cancers, 7/14 (50%) cases were PD-L1-positive in the vulvar site. Among non-HPV-related cancers, 53/87 (60.9%) cases were PD-L1-positive in the vulvar site.

The intensity of immune infiltrate, evaluated with CD3 immunostaining, was moderate or intense in about 80% of non-metastatic vulvar cancers (Group A) and 95% of metastatic vulvar cancers (Group B). In the latter, the proportion of vulvar tumors with moderate immune infiltrate was higher (66% vs. 43.4%; *p* = 0.05), while the proportion of vulvar tumors with slight immune infiltrate or intense immune infiltrate was lower, although this did not reach significance (6.4% vs. 18.9% and 27.7% vs. 37.7%; *p* = 0.05). To further analyze the tumoral immune microenvironment, we investigated the relation between PD-L1 expression and the intensity of immune infiltrate in the vulvar site. Among PD-L1-negative tumors (*n* = 40), despite cancer spread to the lymph nodes, 7/40 (17.5%) showed a slight immune infiltrate, 21/40 (52.5%) showed moderate immune infiltrate, and 12/40 (30%) showed intense immune infiltrate. Among PD-L1-positive tumors (*n* = 60), only 6/60 (10%) were slightly infiltrated by immune cells, while the other percentages were both higher (55% moderate and 35% intense immune infiltrate).

### 3.5. Mismatch Repair Proteins

In our analysis, all tested samples showed retained immunoexpression of MMR proteins (MSH2, MSH6, MLH1, and PMS2); therefore, these markers were excluded from all subsequent correlation analyses.

### 3.6. Hormonal Environment

In our analysis, only two tested samples (belonging to Group B) showed ER expression in at least 1% of tumoral cells; similarly, only three tested samples (belonging to Group A) showed PR expression in at least 1% of tumoral cells. For these reasons, these markers were excluded from all subsequent correlation analysis.

### 3.7. Neoangiogenesis

Only 14/101 (13.9%) tumors showed a strong–moderate diffuse expression of VEGF. There was no significant difference between expression in non-metastatic vulvar cancers (Group A, strong–moderate diffuse expression 15.1%) and metastatic vulvar cancers (Group B, strong–moderate diffuse expression 12.5%). A similar quote of strong–moderate diffuse expression was found in the LNs (15.9%). Similarly, tumor vulvar site vascularization did not seem to differ significantly between non-metastatic and metastatic cancers; in fact, the median of the MVDratio was similar in the two groups (1.5 (q ¼–q ¾ = 1.03–2.14) vs. 1.4 (q ¼–q ¾ = 1.08–2.34)).

### 3.8. Statistical Analysis

All the biomarkers were unable to determine significant Kaplan–Meier OS and DFS survival curves among all patients. Nevertheless, through a GLM, we found that p53 and PD-L1 showed a significant association with nodal metastasis. Odds Ratio (OR) for p53 mutation was 4.26 (CI 95% = 1.14–15.87, *p* = 0.03); OR for PD-L1 positivity was 2.68 (CI 95% = 1.0–7.19, *p* < 0.05) (Table 3).

### 3.9. Cluster Analysis

**Figure 2:**
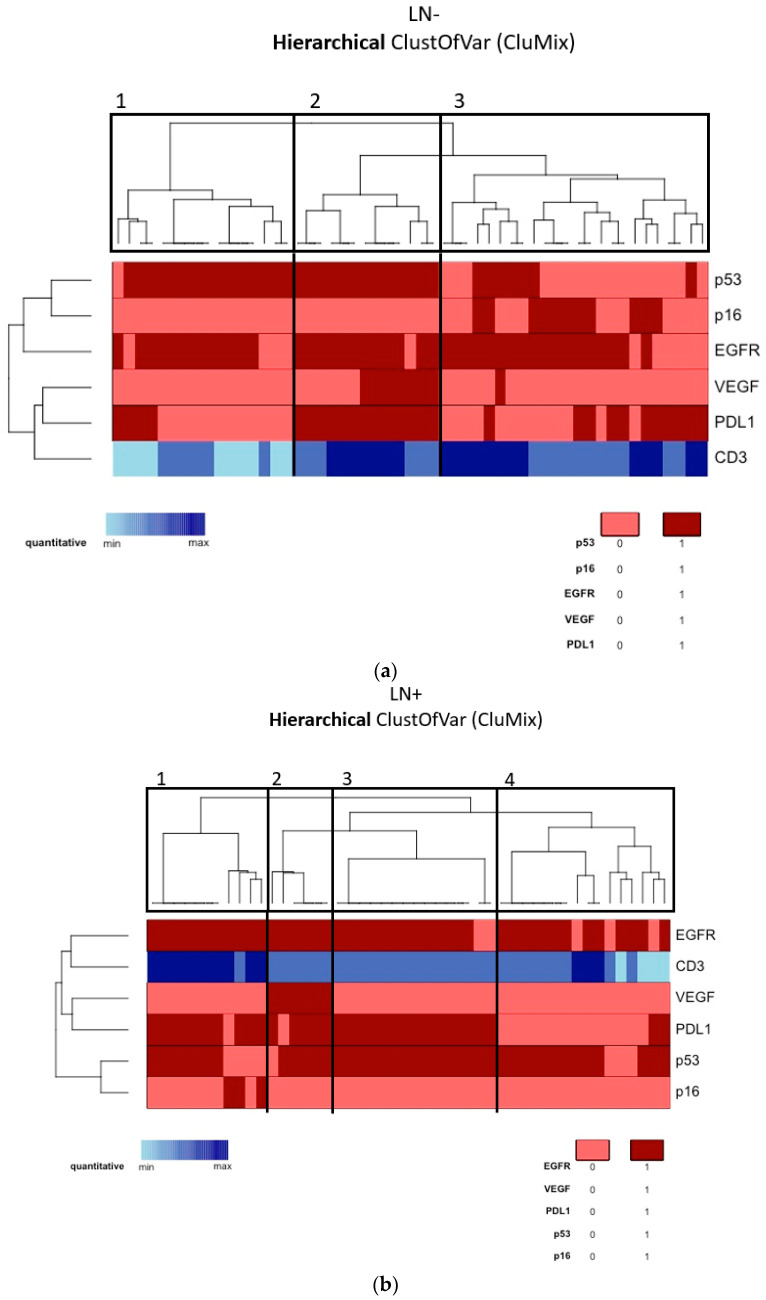
(**a**) Cluster analysis of the molecular profile of the vulvar site among Group A (lymph-node-negative). (**b**) Cluster analysis of the molecular profile of the vulvar site among Group B (lymph-node-positive).

According to the tumor molecular profile of the vulvar site, a cluster analysis was performed through the “CluMix R package”, maintaining the division of our patients into two populations on the basis of nodal status, which is the main known prognostic factor. Due to the lack of many MVD control values, CD31 was excluded from cluster analysis. Moreover, for the abovementioned reasons, MMR proteins, HER2/neu, ER, and PR were also excluded. Among the non-metastatic tumors (Group A, Figure 2a), we identified three main clusters, respectively containing 16, 13, and 24 patients. In the metastatic tumors (Group B, Figure 2b), we identified four main clusters, respectively containing 11, 6, 15, and 16 patients. In this way, in Group A we could distinguish: (1) an HPV-independent cluster showing p53 mutation and EGFR-H (target: EGFR); (2) an HPV-independent cluster showing p53 mutation, EGFR-H, strong VEGF expression, and PD-L1 positivity together with consistent immunocyte infiltration (target: EGFR, VEGF, PD-L1); (3) an HPV-associated cluster showing p53-wild type, EGFR-H, and a certain amount of PD-L1 positivity (target: EGFR, PD-L1). Similarly, in Group B we could say that the majority of tumors were HPV-independent and p53-mutated, and we could distinguish the following clusters: (1) EGFR-H and PD-L1 positivity together with consistent immunocyte infiltration (target: EGFR, PD-L1); (2) strong VEGF expression (target: EGFR, PD-L1, VEGF); (3) similar to the first but less infiltrated by TILs; (4) EGFR-H (target: EGFR). Kaplan–Meier OS and DFS survival curves were not significantly different among clusters in Group A and Group B.

**Figure 3:**
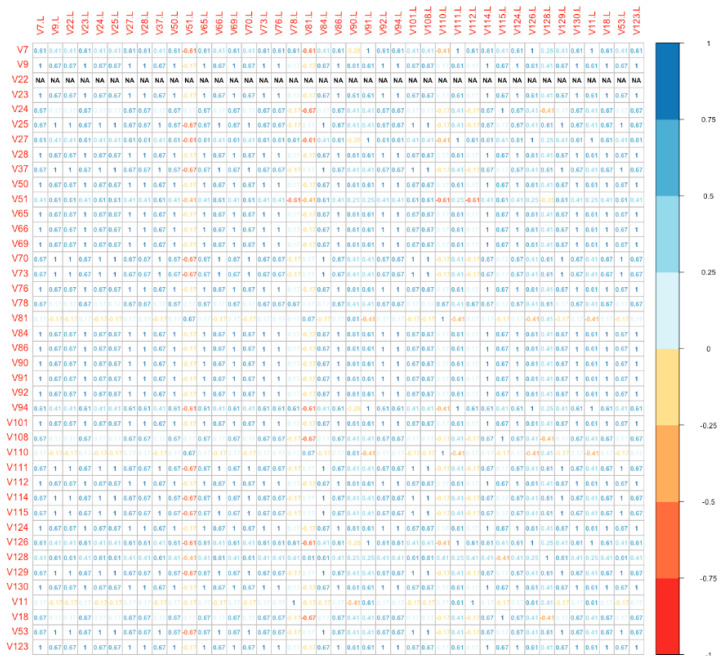
Distance plot (vulvar site vs. lymph nodal site).

Finally, we used a distance plot to compare directly the global molecular profile of each vulvar site with the correspondent lymph nodal metastatic site (among Group B). The plot is shown as a bidimensional matrix with correspondent specimens disposed on the axes. With this model, we found that the IHC panel was significantly different in 18/41 (43.9%) metastatic lymph nodes compared to the primitive vulvar tumor (Figure 3). Numbers on the diagonal are a measure of the similarity between statistical units (molecular “fingerprint” of the samples), which we have considered relevant if >0.7.

## 4. Discussion

The present study provides a detailed IHC analysis on the main biological markers involved both in pathogenesis and potential therapeutic management of VSCC.

Being a monocentric study, a weak point is the unavoidable limited number of patients and the poor representation of p16-positive disease in both study groups, which did not allow us to highlight statistically significant differences in prognosis. Another weakness is the retrospective design, which also limited tissue samples availability and clinical data retrieval.

However, one of the major strengths of the study is the extensive literature and bibliographic research preparatory to the careful selection of the IHC panel applied [26]. Additional points are that: the entire panel of analyses were conducted at a single center and reviewed by a team of experienced pathologists; and patients’ management was always discussed by a dedicated multidisciplinary tumor board (the Gemelli Vul.Can MDT), optimizing the therapeutic choices and reducing as much as possible the multiple variables—other than tumor-dependent ones—that may affect the outcome.

To the best of our knowledge, despite the rather limited monocentric series, this is one of the studies with the largest number of samples assessed for a rich panel of IHC markers; moreover, the IHC expression was compared between the primary tumor and the lymph node metastasis, whenever both were available.

In addition, data clustering represents a first step toward identifying specific groups of patients potentially susceptible to one or more target therapies.

The vast majority of tumors tested in our series (86.1%) were p16-negative and thus HPV-independent. This could be due to the high median age at diagnosis (78 years), as in older patients the prevalent pathogenic pathway is the progression of dVIN to VSCC [28,33]. Our findings confirm that patients with HPV-related vulvar cancer generally show regional confined disease, as p16-positive tumors were significantly more prevalent in non-metastatic patients (Group A). These data are in line with a recent meta-analysis by Zhang et al. (a total of 33 studies and 7721 subjects included), which concluded that HPV-positive vulvar cancer is associated with better OS (HR = 0.64, CI 95%: 0.47–0.87) and recurrence-free survival (HR = 0.66, CI 95%: 0.45–0.97) compared with its HPV-negative counterpart [38]. In accordance with previously published data [39], our study confirms the role of p53 overexpression as negative prognostic predictor, significantly associated with nodal metastases (OR 4.26; CI 95% = 1.14–15.87, *p* = 0.03).

Moreover, the majority of tested cases (82.2%) were p16-positive or p53-mutated in a mutually exclusive way. Additionally, there was a small cohort of p16-positive tumors that also showed p53 mutation (3/14 cases−1.4%), making it very difficult to perform a specific survival analysis; the fraction of p53-mutated tumors among HPV-associated ones varies widely in the literature from 8% to 46% [40,41,42,43,44].

Based on currently available data, the prognosis of VSCC is mainly influenced by the stage of disease at diagnosis and by the presence of lymph node metastases [45]. The 3-year overall survival drops from 90% in node-negative to 56% in node-positive patients [46]. In view of the rarity of this disorder and due to the small number of dedicated randomized clinical trials, in cases of advanced or metastatic disease the prognosis is often poor [47]. In fact, standard chemotherapy did not show relevant survival improvement in the available trials, with the cost of extremely heavy toxicity in cases of combination of multiple agents [47,48,49].

At present, no specific target treatments are available for advanced, metastatic, or recurrent vulvar carcinomas [14,50]. Therefore, the clinical management of these patients quite often represents a challenge. Furthermore, the most promising molecules are selected from those effective in other disorders, such as head and neck or cervical cancer. In particular, the attention is mainly focused on anti-EGFR drugs, immune checkpoint inhibitors (ICIs), and anti-VEGF drugs.

Regarding EGFR status, in the present study we observed no statistically significant association between high EGFR IHC expression, patients’ survival, and nodal metastases.

These data partially differ from those in the literature: (1) Johnson et al. found a correlation with worse DFS in those patients with high IHC EGFR expression [51]; (2) In another study, conducted on 51 vulvar cancers, EGFR gene amplification seemed to characterize a subgroup of HPV-independent tumors linked to decreased survival [52]. Oonk et al., on the contrary, showed how the probability of negative nodes decreased only from 13% to 6% if EGFR expression was considered in addition to classical histopathological parameters, thus losing importance as an adjunctive prognostic factor [53].

In our series, a high percentage of EGFR-H tumors was found. In detail, 79.2% of Group A tumors, 89.6% of Group B tumors, and 95.5% of Group B nodal metastases demonstrated high levels of EGFR immunoexpression. Overall, 84% of vulvar samples were EGFR-H. This percentage is higher than previous studies; in particular, in one of the most important works on EGFR IHC expression in vulvar cancers [53], positive staining for EGFR was observed in 68% of the tumors. As stated in our results, a similar distribution of EGFR immunostaining was observed between HPV and non-HPV-related cancers, so underrepresentation of p16-positive VSCC cases in our study may not be justified. IHC interpretation also seems to be the same, based on a four-point scale for EGFR and a final dichotomous interpretation (0 and 1 = negative; 2 and 3 = positive). The only difference lies in the antibody clone (clone 31G7 vs. clone E30 in our study), and this could explain the approximately 15% higher expression in our series. These data may have important therapeutic implications, since anti-EGFR drugs, such as *cetuximab*, *geftinib*, and *erlotinib*, are increasingly proposed for vulvar cancer patients [54]. In particular, *erlotinib* is actually considered in the current NCCN vulvar cancer guidelines (category of evidence 2B) for advanced/metastatic/recurrent disease, but independently from molecular expression [14].

Moreover, ICIs, such as pembrolizumab, have recently been included in NCCN guidelines as a recommended second-line option for PD-L1-positive advanced or recurrent/metastatic vulvar cancer [14]. Current opinion is that there might be a place for ICIs in vulvar cancer treatment, especially in combination with radiotherapy [55]. Results from the KEYNOTE-826 trial, recently published, show that the progression-free and overall survival were significantly longer with pembrolizumab than with placebo among patients with persistent, recurrent, or metastatic cervical cancer who were also receiving chemotherapy with or without bevacizumab [56]. In particular, PD-L1 expression was measured according to the combined positive score, defined as the number of PD-L1-staining cells (tumor cells, lymphocytes, and macrophages) divided by the total number of viable tumor cells, multiplied by 100. In this trial, the benefit of pembrolizumab relative to that of placebo appeared to increase with increasing PD-L1 expression (combined positive score <1 vs. 1 to <10 vs. ≥10). In our study, PD-L1 evaluation was performed for tumor cells but not for tumor-infiltrating immune cells. The expression of PD-L1 was scored as a percentage of tumor cells with positive PD-L1 membranous staining of any intensity, and analyses were performed using a threshold of greater than or equal to 5%, as we previously provided for vulvar Paget’s disease [31] and as reported in other pre-clinical studies on VSCC [57,58]. PD-L1-positive tumors were 52.8% (28/53) and 66.7% (32/48) in non-metastatic (Group A) and metastatic (Group B) patients, respectively. Moreover, 60% of metastatic lymph nodes were PD-L1 positive, and a rise in expression was observed in five cases. Therefore, our results seem to further support the use of ICIs in vulvar cancer treatment and, as reported in the KEYNOTE-826 trial for cervical cancer patients, the presence of PD-L1 expression could influence the response to treatment; in any case, this remains an hypothesis to be confirmed in specific clinical trials on VSCC patients. Regarding the correlation between PD-L1 expression and HPV status or prognosis, conflicting data are currently available in the literature [55,57,58,59,60,61]. In our study, we observed no statistically significant correlations between PD-L1 and p16 IHC expression. However, in our series, PD-L1 positivity was significantly associated with nodal metastasis (OR 2.68; CI 95% = 1.0–7.19, *p* < 0.05), thus supporting its role as a negative prognostic factor.

Regarding the immune microenvironment, the expression of CD3 in T cells was evaluated to determine TILs infiltration. Our study demonstrated a consistent T-cell-mediated (CD3+) immune response in the vast majority of cases. However, we did not find any correlation between PD-L1 expression and CD3+ tumor-infiltrating lymphocytes.

Additionally, in our study a small percentage (13.9%) of cancers showed a strong or moderate and diffuse IHC staining for VEGF, without any difference between non-metastatic and metastatic group. Moreover, the tumor vulvar site microvascularization did not seem to differ significantly between non-metastatic and metastatic cancers, with a similar median of the MVD ratio in the two groups (1.5). Therefore, our data suggest that a minority of VSCC patients would benefit from a therapy with monoclonal antibodies against VEGF (bevacizumab), which currently represents a therapeutic option for recurrent/metastatic cervical cancer approved by the FDA [62,63]. This therapeutic option for VSCC is also derived from the results from studies involving cervical cancer, which suggest a chance of improved survival. Further correlation studies regarding the HPV-related versus HPV-independent pathway would be interesting in order to select subgroups of patients more responsive to this target treatment. Currently, NCCN guidelines suggest considering bevacizumab in combination with paclitaxel and cisplatin for the treatment of relapsed or metastatic disease, even if with a level of evidence of 2B [14].

Lastly, cluster analysis was an intuitive way to define possible molecular “fingerprints” for subsets of patients that could benefit from specific target therapies.

In detail, we clustered Group A (non-metastatic) in three subclasses: (1) EGFR represented a biomarker commonly expressed by all the three different clusters; moreover, (2) non-HPV-related tumors showed high levels of neoangiogenesis and immune response markers, while (3) HPV-related tumors were less vascularized and expressed variable levels of PD-L1.

In Group B, we were able to define four clusters. Among all Group B clusters, EGFR was commonly expressed, and most tumors were non-HPV-related and p53-mutated. Therefore, according to the abovementioned findings, anti-EGFR drugs could be taken into account for almost all metastatic patients, for which reason it is important to identify additional systemic treatments to improve prognosis. Furthermore, we identified two clusters that could benefit from anti-PD-L1 drugs (cluster 1 and 3) and another cluster that could also benefit from anti-VEGF drugs (cluster 2).

Starting from this classification, it emerges on one hand that there is no clear distribution of IHC expression for the different markers between the clusters. On the other hand, the simultaneous presence of more than one molecular target could open the chance for combination therapies versus single agent treatment.

Moreover, the correlation plot analysis demonstrated that it is mandatory to repeat the panel in the metastatic site to identify eventual changes of marker expression or possible therapeutic targets.

Finally, despite all limits, the present study contributes to lay the groundwork for clinical trials aimed at the use of target therapies. Further studies are needed to consolidate the obtained results and should be based on a multicenter conception, to allow the inclusion of an adequate number of cases to expand the actual knowledge.

## 5. Conclusions

Our results support a potential role of ICIs and anti-VEGF and anti-EGFR drugs in specific subsets of VSCC patients, especially with the worse prognosis (metastatic, HPV-independent). A more favorable prognosis for HPV-related tumors and the association of p53-mutated and PD-L1-positive tumors with nodal metastasis are also confirmed.

We suggest routinely performing a small panel including EGFR, VEGF, PDL1, p16, and p53 in both primary tumor and nodal/distant metastasis: it is highly recommended to repeat the panel in the metastatic site to identify changes in marker expression and thus a possible gain or loss of therapeutic targets. Prospective and multicenter studies are needed to consolidate these results.

## Figures and Tables

**Table 1:** Patients’ characteristics.

Patients’ Characteristics	All (n. 101)	Group A (n. 53)	Group B (n. 48)	*p*-Value
		Lymph-Node-Negative	Lymph-Node-Positive	
Age				
Median (range)	78 (48–96)	81 (48–96)	75.5 (56–92)	0.01
BMI				
Median (range)	26.3 (15.8–47.3)	25.05 (15.8–42.2)	29.7 (19–47.3)	0.004
Comorbidities				
None	18 (17.8%)	12 (22.6%)	6 (12.5%)	0.18
CV (hypertension and/or cardiovascular disease)	25 (24.8%)	9 (17%)	16 (33.4%)	
Diabetes	7 (6.9%)	3 (5.7%)	4 (8.3%)	
Multiple *	51 (50.5%)	29 (54.7%)	22 (45.8%)	
Tumor characteristics (primary site)				
Site				
Anterior	55 (54.4%)	27 (51.0%)	28 (58.4%)	0.91
Central	10 (9.9%)	6 (11.3%)	4 (8.3%)	
Posterior	10 (9.9%)	6 (11.3%)	4 (8.3%)	
Lateral	26 (25.8%)	14 (26.4%)	12 (25%)	
Focality				
Unifocal	98 (97%)	52 (98.1%)	46 (95.8%)	0.6
Multifocal	3 (3%)	1 (1.9%)	2 (4.2%)	
Type of vulvar surgery				
Partial vulvectomy	30 (29.7%)	14 (26.4%)	16 (33.3%)	0.78
Radical vulvectomy	49 (48.5%)	27 (50.9%)	22 (45.9%)	
Ultraradical vulvectomy	22 (21.8%)	12 (22.7%)	10 (20.8%)	
Inguino-femoral surgery ^				
SLNB	56 (29.7%)	44 (46.3%)	12 (12.9%)	°
SLNB + Lymphadenectomy	79 (42.1%)	42 (44.2%)	37 (39.8%)	
Lymphadenectomy	53 (28.2%)	9 (9.5%)	44 (47.3%)	
Side				
Monolateral	6 (5.9%)	5 (10.0%)	1 (2.1%)	
Bilateral	91 (90.1%)	45 (90.0%)	46 (97.9%)	°
Grading				
G1	9 (8.9%)	9 (17%)	0 (0%)	0.001
G2	83 (82.2%)	42 (79.2%)	41 (85.4%)	
G3	9 (8.9%)	2 (3.8%)	7 (14.6%)	
Depth of invasion				
Median (range), mm	6 (0.9–25)	6 (0.9–25)	7 (0.9–25)	0.001
Maximum tumor diameter				
Median (range), mm	35 (4–105)	30 (5–80)	39.5 (4–105)	0.18
<2 cm	25 (24.8%)	17 (32.1%)	8 (16.7%)	
2–4	28 (27.7%)	12 (22.6%)	16 (33.3%)	
≥4 cm	48 (47.5%)	24 (45.3%)	24 (50%)	
LVSI				
Yes	32 (31.7%)	2 (3.8%)	30 (62.5%)	<0.0001
No	69 (68.3%)	51 (96.2%)	18 (37.5%)	
Number of excised nodes				
SLNs				0.36
Total median number (range)	3 (1–16)	4 (1–10)	3 (1–16)	
Lymphadenectomy				
Total median number (range)	12 (1–43)	6 (1–43)	14 (5–31)	
Extracapsular spread				
Present	10 (10.2%) ^a^	0 (0%) ^b^	9 (18.8%) ^c^	°
Absent	88 (89.8%)	50 (100%)	39 (81.2%)	
FIGO Stage				
I	49 (48.5%)	49 (92.5%)	0 (0%)	°
II	4 (4%)	4 (7.5%)	0 (0.0%)	
III	43(42.6%)	0 (0%)	43 (89.6%)	
IV				
A	0 (0%)	0	0	
B	5 (4.9%)	0 (0%)	5 (10.4%)	
Adjuvant treatment				
RT	53 (52.5%)	23 (43.4%)	30 (62.5%)	0.018
RT-CT	3 (3%)	1 (1.9%)	2 (4.2%)	
CT	2 (2%)	0 (0%)	2 (4.2%)	
None	43 (42.5%)	29 (54.7%)	14 (29.1%)	
FU				
DOD (%)	12 (11.9%)	2 (3.8%)	10 (20.8%)	0.006
OS Median (range, months)	17 (1–67)	22 (1–54)	13.5 (1–67)	
DFS median (range, months)	14 (0.25–67)	20 (1–52)	9 (0.25–67)	

Group A = lymph-node-negative; Group B = lymph-node-positive. Abbreviations: BMI: body mass index; SLNB: sentinel lymph node biopsy; SLNs: sentinel lymph nodes; LVSI: lymphovascular space invasion; RT: radiotherapy; CT: chemotherapy; DOD: death of disease; OS: overall survival; DFS: disease free survival. * Multiple: includes one of the above plus other comorbidities such as depression, thyroid disease, chronic venous insufficiency etc. ^a^ Data available for 98/101 patients; ^b^ Data available for 50/53 patients; ^c^ Data available for 48/48 patients. ° *p*-Value not considered given the selection of the two groups. ^^^ Data stratified per groin—n°3 patients did not undergo groin surgery.

**Table 2:** Immunohistochemical panel.

Biomarkers	T All	T Group A	T Group B	*p*-Value *	N Group B
	*n* = 101	*n* = 53	*n* = 48		*n* = 48
p16					
Negative (HPV-independent)	87 (86.1%) ^a^	42 (79.2%) ^b^	45 (93.8%) ^c^	0.04	40 (90.9%) ^e^
Positive (HPV-associated)	14 (13.9%)	11 (20.8%)	3 (6.2%)		4 (9.1%)
p53					
Wild type	26 (25.7%) ^a^	18 (34.0%) ^b^	8 (16.7%) ^c^	0.06	6 (13.6%) ^e^
Mutated	75 (74.3%)	35 (66.0%)	40 (83.3%)		38 (86.4%)
PD-L1 (% positive cells)					
Median (q_1/4_–q_3/4_) (range)	5 (1–20) (0–90) ^a^	5 (0–10) (0–90) ^b^	10 (2.75–30) (0–90)	0.22	8 (0–25) (0–100) ^d^
Negative	41 (40.6%)	25 (47.2%)	16 (33.3%)		18 (40%)
Positive (> 5%)	60 (59.4%)	28 (52.8%)	32 (66.7%)		27 (60%)
TILs (CD3)					
Slight	13 (13.0%) ^f^	10 (18.9%) ^b^	3 (6.4%) ^g^	0.05	-
Moderate	54 (54.0%)	23 (43.4%)	31 (66.0%)		
Intense	33 (33.0%)	20 (37.7%)	13 (27.6%)		
MSH2					
Negative	0 (0%) ^a^	0 (0%) ^b^	0 (0%) ^c^	1	0 (0%) ^d^
Positive	101 (100%)	53 (100%)	48 (100%)		45 (100%)
MSH6					
Negative	0 (0%) ^a^	0 (0%) ^b^	0 (0%) ^c^	1	0 (0%) ^d^
Positive	101 (100%)	53 (100%)	48 (100%)		45 (100%)
MLH1					
Negative	0 (0%) ^a^	0 (0%) ^b^	0 (0%) ^c^	1	0 (0%) ^d^
Positive	101 (100%)	53 (100%)	48 (100%)		45 (100%)
PMS2					
Negative	0 (0%) ^a^	0 (0%) ^b^	0 (0%) ^c^	1	0 (0%) ^d^
Positive	101 (100%)	53 (100%)	48 (100%)		45 (100%)
EGFR					
Low expression	16 (15.8%) ^a^	11 (20.8%) ^b^	5 (10.4%) ^c^	0.25	2 (4.5%) ^e^
High expression	85 (84.2%)	42 (79.2%)	43 (89.6%)		42 (95.5%)
HER2/neu					
Negative	99 (98%) ^a^	52 (98.1%) ^b^	47 (98%) ^c^	1	42 (93.3%) ^d^
Overexpressed/amplified	2 (2%)	1 (1.9%)	1 (2%)		3 (6.7%)
ER (% positive cells)					
Median (q_1/4_–q_3/4_) (range)	0 (0–0) (0–40) ^a^	0 (0–0) (0–0) ^b^	0 (0–0) (0–40) ^c^	0.22	0 (0–0) (0–50) ^d^
Negative	99 (98%)	53 (100%)	46 (95.8%)		44 (97.8%)
Positive (≥1%)	2 (2%)	0 (0%)	2 (4.2%)		1 (2.2%)
PR (% positive cells)					
Median (q_1/4_–q_3/4_) (range)	0 (0–0) (0–15) ^a^	0 (0–0) (0–15) ^b^	0 (0–0) (0) ^c^	0.24	0 (0–0) (0–0) ^d^
Negative	98 (97.1%)	50 (94.3%)	48 (100%)		45 (100%)
Positive (≥1%)	3 (2.9%)	3 (5.7%)	0 (0%)		0 (0%)
VEGF					
Absent, Weak, Moderate % <10	87 (86.1%) ^a^	45 (84.9%) ^b^	42 (87.5%) ^c^	0.77	37 (84.1%) ^e^
Moderate % ≥10, Strong	14 (13.9%)	8 (15.1%)	6 (12.5%)		7 (15.9%)
MVD					
Mean MVD—median				0.37	
(q_1/4_–q_3/4_) (range)	28 (20–36) (5–63) ^a^	28 (20–32) (5–63) ^b^	29 (19.5–40) (7–57)		-
Max MVD—median					
(q_1/4_–q_3/4_) (range)	35 (25–50) (5–70) ^a^	30 (25–45) (5–70) ^b^	40 (30–54.25) (10–70) ^c^		-
MVD sane tissue—median					
(q_1/4_–q_3/4_) (range)	20 (11–25) (5–60) ^h^	20 (10–25) (5–60) ^i^	15 (13.5–25) (5–60) ^l^		-
MVD ratio—median					
(q_1/4_–q_3/4_) (range)	1.5 (1.06–2.25) (0.4–9.4) ^h^	1.5 (1.03–2.14) (0.4–9.4) ^i^	1.4 (1.08–2.34) (0.4–4.5) ^l^		-

Group A = lymph-node-negative; Group B = lymph-node-positive. Data are median (q1/4–q3/4) (range) or n (%), as appropriate. Percentages are calculated over the total of available data. * *p*-Value of T Group A vs. T Group B. Abbreviations: T Group A: vulvar site from non-metastatic tumor; T Group B: vulvar site from metastatic tumor; N Group B: metastatic lymph nodal site; PD-L1: programmed death-ligand 1; MSH2: mutS protein homolog 2; MSH6: mutS protein homolog 6; MLH1: mutL protein homolog 1; PMS2: postmeiotic segregation increased 2; ER: estrogen receptor; PR: progesterone receptor, HER2/neu: human epidermal growth factor receptor 2; EGFR: epidermal growth factor receptor; TILs: tumor infiltrating lymphocytes; VEGF: vascular endothelial growth factor; MVD: microvessel density. ^a^ Data available for 101/101 patients; ^b^ data available for all patients (53/53); ^c^ data available for 48/48 patients; ^d^ data available for 45/48 patients; ^e^ data available for 44/48 patients; ^f^ data available for 100/101 patients; ^g^ data available for 47/48 patients; ^h^ data available for 91/101 patients; ^i^ data available for 48/53 patients; ^l^ data available for 43/48 patients.

**Table 3:** Generalized linear model for biomarkers. Odds for lymph node metastasis in the mutant (exposed) over the non-mutant (not exposed).

Biomarkers	Odds Ratio	Confidence Interval at 95%	
p53	4.2686 *	1.1478	15.8750
EGFR	2.8069	0.7215	10.9206
p16	0.4105	0.0806	2.0924
PDL1	2.6857 *	1.0024	7.1952
CD3	1.5377	0.7499	3.1531
VEGF	0.2691	0.0615	1.1767
CD31	1.0044	0.3908	2.5811

* *p* < 0.05. The table shows the estimates for the univariate regression for each predictor in the current study. The estimate shows the odds ratio variation per unitary increase of each marker.

## Data Availability

Clinical data were collected using Research Electronic Data Capture (REDCap) tool and managed according to privacy regulations.

## Supplementary Materials

The following are available online at https://www.mdpi.com/article/10.3390/cancers13246373/s1, Table S1: Antibodies used for immunohistochemical reactions, Figure S1: Representative examples of immunohistochemical staining of PD-L1, CD3, VEGF, EGFR, CD31, HER2/neu. Figure S2: Representative examples of immunohistochemical staining of HPV-related and HPV-independent VSCC.
