# Supplementary material for: The Vulvar Immunohistochemical Panel (VIP) Project: Molecular Profiles of Vulvar Squamous Cell Carcinoma
Source: Cancers (Basel). 2021 Dec 19;13(24):6373. doi: 10.3390/cancers13246373 (PMC8699435; doi:10.3390/cancers13246373)
Supplement: Supplementary file 1 [file cancers-13-06373-s001.zip › cancers-1427118-supplementary.pdf]

cancers

MDPI

Supplementary Materials

The Vulvar Immunohistochemical Panel (VIP) Project: Molecular Profiles of Vulvar Squamous Cell Carcinoma

Giorgia Garganese , Frediano Inzani, Simona Maria Fragomeni, Giulia Mantovani, Luigi Della Corte , Alessia Piermattei, Angela Santoro, Giuseppe Angelico, Luciano Giacò, Giacomo Corrado, Anna Fagotti, Gian Franco Zannoni and Giovanni Scambia

Table S1. Antibodies used for immunohistochemical reactions.

Antibody	Clone	Antibody Supplier	Detection	Instrument
anti-ER	SP1	Roche/Ventana, Tucson, AZ	UltraView Universal DAB Detection Kit	BenchMark ULTRA (Roche/Ventana)
anti-PR	1E2			
anti-HER2	4B5			
anti-p53	Bp53-11			
anti-CD3	2GV6			
anti-p16	CINtec® Histology Kit			
PD-L1	SP263		OptiView DAB IHC Detection Kit	
anti-MLH1	M1			
anti-MSH2	G219-1129			
anti-MSH6	SP93			
anti-PMS2	A16-4			
anti-CD31	JC70A	LEICA	BOND™ Polymer Refine Detection System	Bond III automated immunostainer (Leica Microsystems, IL)
anti-VEGF	VG1	DAKO/AGILENT		
anti-EGFR	E30	DAKO/AGILENT		

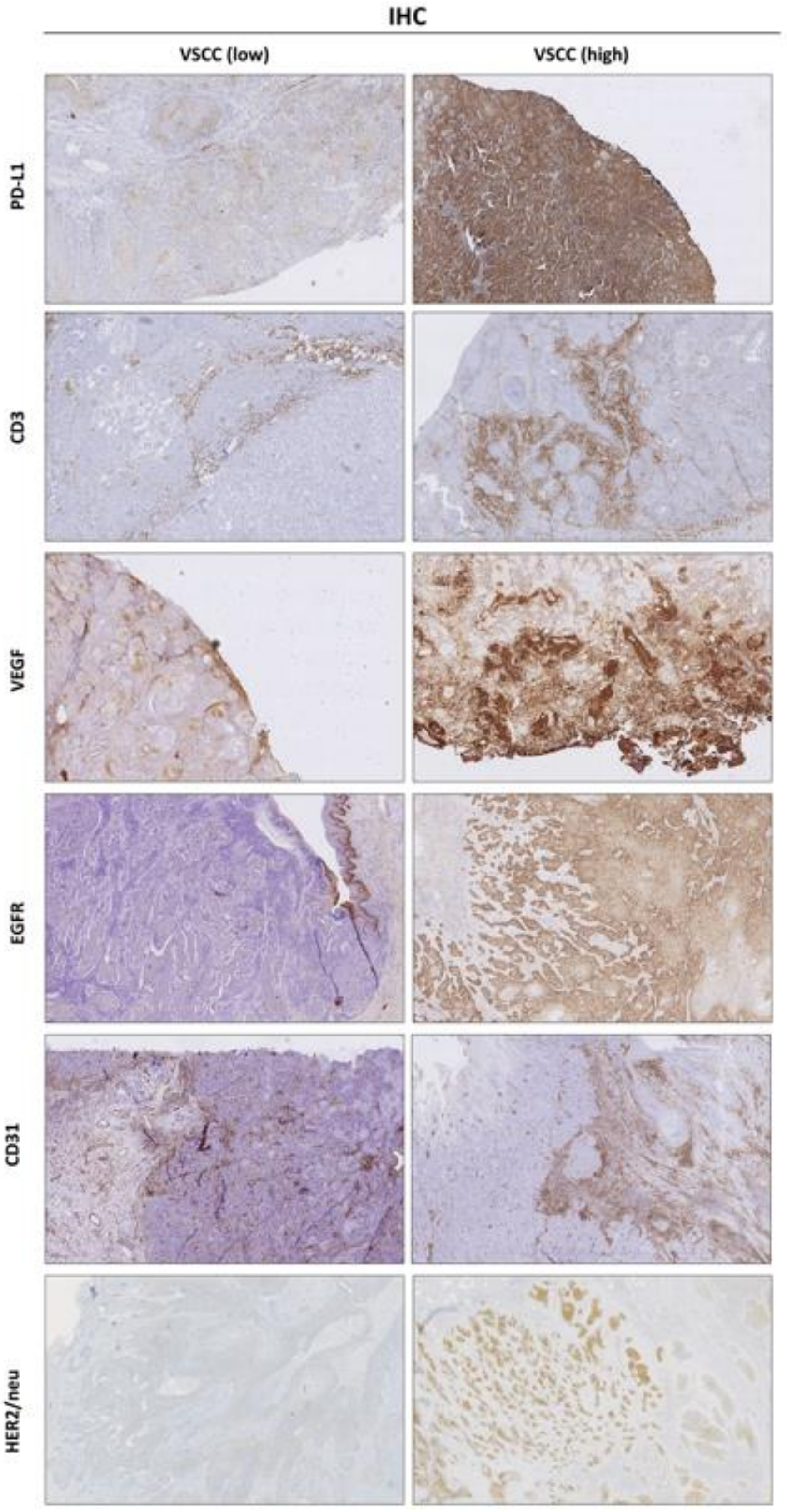
IHC
VSCC (low)
VSCC (high)
PD-L1
CD3
VEGF
EGFR
CD31
HER2/neu

Figure S1. Representative examples of low immunohistochemical staining of PD-L1 (60x), CD3 (40x),VEGF (50x), EGFR (60x), CD31 (70x), HER2/neu (100x) (left panel) and high immunohistochemical staining of PD-L1 (60x), CD3 (70x),VEGF (40x), EGFR (70x) , CD31 (50x), HER2/neu (60x) (right panel).

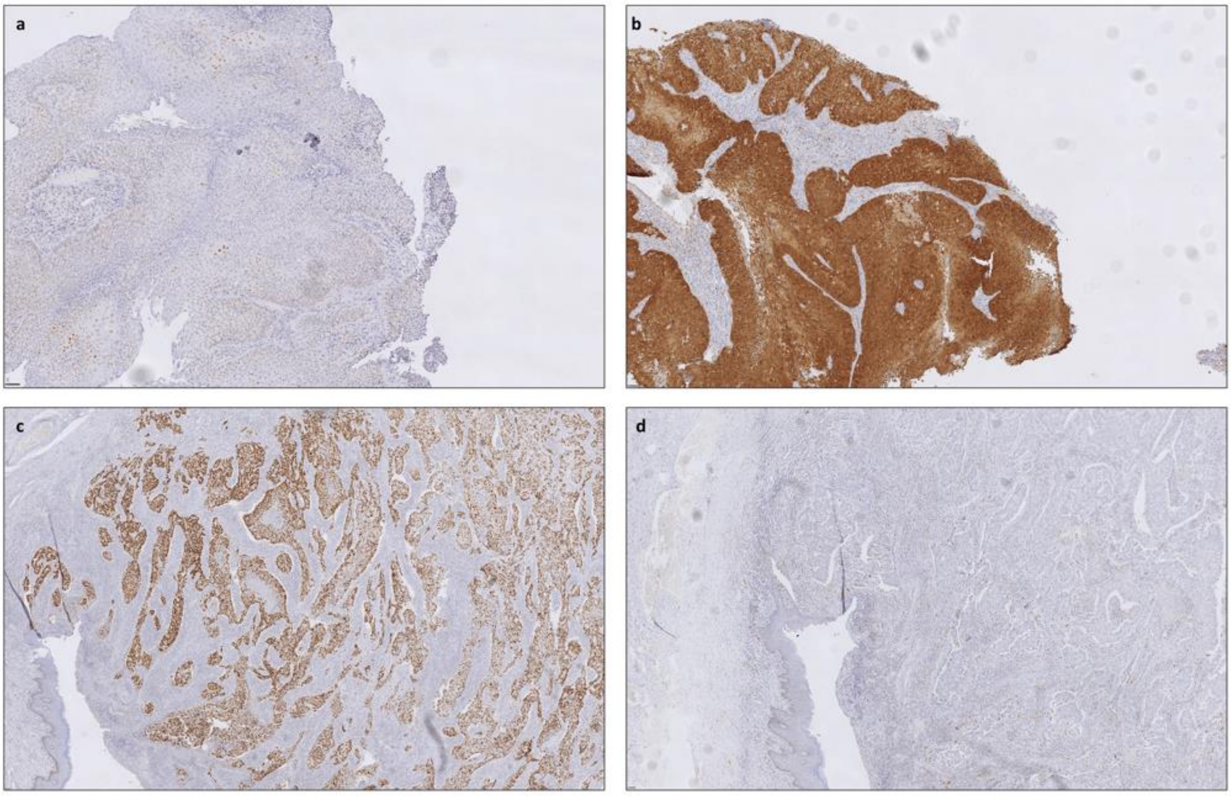

Figure S2. Representative examples of immunohistochemical staining of HPV-related (upper panels) and HPV-independent (lower panels) VSCC. (**a**) p53-wild type (140x), (**b**) p16 INK4a (score 2) (100x), (**c**) p53-mutated (50x), (**d**) p16 ^{INK4a} (score 0) (80x).
